# When Does Episodic Memory Contribute to Performance in Tests of Working Memory?

**DOI:** 10.5334/joc.311

**Published:** 2023-08-03

**Authors:** Klaus Oberauer, Lea M. Bartsch

**Affiliations:** 1 Department of Psychology, University of Zurich, Switzerland

**Keywords:** Working memory, episodic memory, serial recall, proactive interference

## Abstract

Both the experimental and the psychometric investigation of the WM capacity limit depend critically on the assumption that performance in our tests of WM reflects that capacity limit to a good approximation. Most tasks to measure WM rely on testing memory after a short time during which participants are asked to maintain information in WM. In these tests, episodic long-term memory is likely to also lay down a trace of the memory set. Therefore, participants can draw on two sources of information when memory is tested, making it difficult to separate the contributions of WM and episodic LTM to the performance on immediate-memory tests. Here we use proactive interference to distinguish between these two sources of remembered information, building on the fact that episodic memory is vulnerable to proactive interference, whereas WM is protected against it. We use a release-from-PI paradigm to determine the extent to which commonly used WM tasks reflect contributions from episodic LTM. We focus on memory for serial order of verbal lists, but also include visual and spatial WM tasks. The results of five experiments demonstrate that although some tasks used to investigate WM are heavily contaminated by episodic LTM, other popular paradigms such as serial and probed recall, and the standard version of the continuous color-reproduction task, are not. Measuring proactive interference can help researchers determine the extent to which WM and episodic LTM contribute to performance in immediate-memory tasks.

Working memory is the mechanism that enables the mind to hold a small set of representations temporarily available for processing. It appears to have a severely limited capacity, as performance in tests of working memory (WM) deteriorates precipitously as the amount of information to be held available is increased (Cowan, 2005; Luck & Vogel, 1997; Oberauer & Kliegl, 2001). Measures of WM capacity are highly correlated with general cognitive ability (Kyllonen & Christal, 1990), and in particular with reasoning ability (Buehner, Krumm, & Pick, 2005; Kane et al., 2004; Süß, Oberauer, Wittmann, Wilhelm, & Schulze, 2002). This strong relationship supports the assumption that WM capacity is a major constraint for the complexity of human reasoning (Halford, Cowan, & Andrews, 2007; Oberauer, Süß, Wilhelm, & Sander, 2007).

Both the experimental and the psychometric investigation of the WM capacity limit depend critically on the assumption that performance in our tests of WM reflects that capacity limit to a good approximation. This might not be the case. Nearly all tasks used to test WM are variants of immediate-memory tests, in which participants are asked to remember a set of stimuli for a few seconds, or tens of seconds. These tasks certainly challenge the ability of WM to maintain the memory set in an accessible state until the test. At the same time, however, episodic long-term memory (eLTM) also lays down a trace of the memory set. Studies of incidental episodic memory show that information that we attend to is encoded into episodic memory even if we don’t intend to remember it (Rock & Gutman, 1981). These conditions are met when people try to encode stimuli into working memory. Hence, there is reason to expect that every information we ask people to maintain for an immediate test is encoded not only into WM but also into eLTM.

When memory is tested, participants can therefore draw on two sources of information, the representations maintained in WM, and the episodic memory representation. Performance in any immediate-memory test must be understood as a combination of contributions of information from these two sources. If the contribution of eLTM is substantial, it helps circumvent the capacity limit of WM, so that when researchers take performance on WM tests as reflections of WM capacity, they could overestimate that capacity.

We can use proactive interference to distinguish between WM and eLTM as the sources of remembered information. Episodic memory is vulnerable to proactive interference: Earlier memories interfere with subsequently acquired memories, and the degree of interference increases with the similarity between the earlier memory contents and the target content (i.e., the content that the person aims to retrieve) (Craik & Birtwistle, 1971; Postman & Keppel, 1977; Watkins & Watkins, 1975). By contrast, WM is protected against proactive interference from episodic memory representations of earlier events (Oberauer, Awh, & Sutterer, 2017; Oberauer & Greve, 2021). Therefore, a test of immediate memory that reflects only WM should show no build-up of proactive interference across trials. However, an immediate memory test that draws substantially on eLTM should show proactive interference across trials.

We have used this rationale to gauge the contribution of eLTM to tests of WM in two previous studies. Bartsch and Oberauer (2023) investigated immediate memory for word-object pairs, a test of the ability to form and maintain temporary bindings between items that is a strong predictor of individual differences in WM capacity and fluid intelligence (Wilhelm, Hildebrandt, & Oberauer, 2013). Proactive interference (PI) between successive trials was varied across experimental sessions: In the low-PI session, participants received pairs randomly composed of elements that were sampled from large pools of words and objects, without repeating a word or an object within the experimental session. The pairs of the high-PI session were combined from small pools of words and objects, such that the same words and objects were used again and again, always in new random pairings. The re-use of the same words and objects fosters proactive interference through cue overload (Watkins & Watkins, 1975): After a number of trials, the word or object used as retrieval cue in the memory test is associated with many other elements in episodic memory, which makes it difficult to selectively access the one associate that is the target on the current trial. For small memory set sizes – two or three pairs – performance did not differ between high or low-PI sessions, but for larger set sizes, performance was worse in the high-PI session. This suggests that with larger set sizes – which challenge the capacity of WM – the memory system starts drawing on information in eLTM to support performance. In the high-PI session, access to eLTM from the current trial is compromised more by proactive interference, leading to poorer performance at larger set sizes than in the low-PI session. Further support for this interpretation comes from two observations. One is that a distractor task in the retention interval, which is assumed to interfere with the contents of WM but not episodic memory, selectively disrupted memory for pairs at small but not large set sizes. The other is that increasing the set size (i.e., the number of pairs to be remembered) led to a substantial drop in performance from two to three to four pairs, but only very little further decline between four and eight, or (in one experiment) even 16, pairs. A relatively constant level of memory accuracy between set sizes from four onward is incompatible with the assumption that performance is constrained by the capacity limit of WM, but it can be explained if we assume that retrieval draws heavily on eLTM at larger set sizes.

A second study used the same approach to reveal the contribution of eLTM to a test of visual WM (Oberauer & Awh, 2022). Participants remembered pictures of everyday objects rendered in a color randomly selected from a color circle, and tried to reproduce each object’s color by selecting it from a color circle. In the low-PI group, objects were sampled from a large pool, so that no object was presented more than once throughout the experiment. In the high-PI group, objects were sampled from a small pool, so that the same objects are repeated frequently across trials, always with a new color. Memory performance did not differ between groups up to set size four, but for larger set sizes, the low-PI group performed better, showing little further increase in the average error of reproduction with increasing set size, whereas the high-PI group continued to make larger errors with larger set sizes. These results support the assumption that at larger set sizes color reproduction relied increasingly on eLTM, rendering it vulnerable to proactive interference.

In the present study we ask to what extent other commonly used tasks for studying WM reflect in part contributions from eLTM, using proactive interference as an empirical signature of eLTM. We are mainly concerned with memory for serial order of verbal lists, because these are the most frequently used tasks for investigating WM. We test memory for serial order in two ways: Serial recall (i.e., recalling the items in the order of presentation), and probed recall (i.e., probing recall of list items by their serial position in the list). Experiments 3, 4, and 5 will also investigate the role of eLTM in a visual and a spatial WM task.

For tests of memory for serial order, we cannot manipulate proactive interference in the same way as we did for object-word pairs and object-color pairs, because we have less control over the retrieval cues. When we ask participants to remember a list of words in their correct order, the retrieval cue for each word is its ordinal position in the list. This is obvious in the probed-recall task, in which memory for each word is tested by probing its list position, either as the ordinal position number (e.g., “what was the third word?”) or as a spatial proxy of the ordinal position (e.g., presenting the list words across a row of frames from left to right, and probing a list position by highlighting a frame). When asking participants to recall a list in forward order, they most likely generate positional retrieval cues themselves, starting recall by re-activating a representation of the first position to cue the first item, then re-activating the second position to cue the second item, and so on. This assumption is shared by the currently most successful models of serial recall (Farrell & Lewandowsky, 2004; Lewandowsky & Farrell, 2008; Oberauer, Lewandowsky, Farrell, Jarrold, & Greaves, 2012). The use of ordinal positions as retrieval cues implies that experiments testing serial-order memory across many trials necessarily create a situation of high proactive interference between trials, because the same set of retrieval cues is used in every trial. After the first few trials, the position cues are already associated with several words, creating substantial cue overload. This cue overload is reflected in protrusion errors in tests of immediate serial recall: When participants erroneously recall words from an earlier trial, these words are most likely recalled in the position in which they had occurred in the earlier trial (Fischer-Baum & McCloskey, 2015; Osth & Dennis, 2015).

To circumvent the problem that we cannot manipulate proactive interference by contrasting unique and repeated retrieval cues, in the present experiments we used a release-from-PI paradigm. Early research has used this paradigm to study proactive interference in tasks that tap primarily episodic memory, such as the Brown-Peterson task, in which encoding of a short list of items is followed by a retention interval filled with a demanding distractor task that is assumed to engage WM. These experiments have shown that proactive interference builds up over about four or five successive trials using similar materials. When the memory material changes from one trial to the next (e.g., from letters to words), proactive interference is substantially reduced (Wickens, 1970). This release from proactive interference shows that interference arises predominantly from the last three to four trials. This conclusion converges with the finding that above-chance protrusions in serial-recall tasks come predominantly from recent earlier trials, up to about four trials back (Fischer-Baum & McCloskey, 2015). The release from proactive interference upon changing to a new class of memory items shows that people use the material class as a retrieval cue, thereby excluding memories from preceding trials that do not match the class of materials studied on the current trial.

A few previous studies have used the release-from-PI paradigm with immediate serial recall tasks. Sanders and Willemsen (1978) presented lists of consonants in pairs of trials, separated by five-minute breaks. The second trial in a pair was recalled more poorly, showing proactive interference from the first trial; the breaks between pairs apparently led to release from proactive interference. Tehan and Turcotte (2002) investigated serial recall of word lists organized into sets of four trials, with breaks of at least two hours between one set and the next. These authors found no evidence for build-up of proactive interference across the four trials of a set. Bunting (2006) studied complex-span tasks in which participants remembered lists of words or of digits, and verified arithmetic equations in between presentation of the list items. The list material – words or digits – changed after every third trial. Across each mini-block of three trials with the same material, recall performance became worse, reflecting the build-up of proactive interference. Performance recovered after changing to the other material, showing release from proactive interference. Oberauer (2022b) reports three experiments in which participants recalled lists of digits, letters, or words, organized into mini-blocks of five trials using the same material before switching to another class of materials for the next mini-block. In one of these experiments there was no evidence for proactive interference; in two others, there was a small decline of performance across the trials of a mini-block. In one of these (Experiment 3), this decline was observed primarily with slow presentation rates.

One further experiment investigated proactive interference in probed recall. Jones and Oberauer (2013) presented lists of five words from five semantic categories across five locations from top to bottom. Recall was cued either by location, or by semantic category. List words were sampled from the same five categories for four successive trials, then the categories were exchanged to enable release from proactive interference. There was evidence for build-up and release from proactive interference with category cues but not with location cues.

In sum, several tasks used to measure working memory are vulnerable to proactive interference across trials at large set sizes. This finding supports the assumption that eLTM is recruited as a backup when the task demand stretches the capacity of WM. However, the evidence for proactive interference is weaker and comparatively inconsistent for tests of immediate serial recall or probed recall, at least in the absence of a concurrent distractor task. Therefore, we ran the present series of experiments to search for more compelling evidence for proactive interference in tests of immediate serial-order memory. Table 1 provides an overview of the present experiments.

**Table 1:** Overview of experiments.


EXPERIMENT	SERIAL-ORDER MEMORY TASK	OTHER TASK	LENGTH OF MINI-BLOCKS	INTER-ITEM INTERVAL (S)	BF_10_ IN FAVOR OF PI IN SERIAL-ORDER MEMORY

1a	Serial Recall	Object-Word Pairs	4	1	0.07

1b	Serial Recall	Object-Word Pairs	4	1	2.2

2a	Probed Recall	Object-Word Pairs	4	1	28.7

2b	Probed Recall	Object-Word Pairs	8	1	0.04

3	Serial Recall	Spatial location reproduction	4	1	0.06

4	Probed Recall	Color reproduction	4	1	0.17

5	Probed Recall	Color reproduction	4	5	0.06


## Experiment 1

In Experiment 1 we compared the build-up and release from proactive interference in two tasks, the WM test for object-word pairs, for which our earlier work has demonstrated proactive interference at larger set sizes (Bartsch & Oberauer, 2023), and serial recall of word lists. In the pairs task, six object-word pairs were to be remembered, and at test, all objects were presented in random order as retrieval cues for the words that had been paired with them. In the serial-recall task, seven words were presented, and at test, participants were asked to retrieve them in forward order. Although forward serial recall does not require explicit cues for each item, we presented participants with the digits from 1–7 at test, representing the respective serial positions, to create comparable test procedures for the two tasks. We chose the set sizes of both tasks so that they unambiguously challenge or exceed WM capacity. For the pairs task, our previous work provided evidence that eLTM contributed to performance for set sizes of four or more pairs (Bartsch & Oberauer, 2023). For serial recall of words, our choice was guided by previous experiments using similar materials and procedures, which showed a steep decline of performance for set sizes exceeding four words, and yielded capacity estimates of about 5 words (Oberauer, 2019). In the first version of the experiment (Experiment 1a), average accuracy in the pairs task was lower than in the serial-recall task. Therefore, we ran the experiment again (Experiment 1b) after reducing the set size of the pairs task to five pairs.

The trials were organized into mini-blocks of four of the same task, using the same set of 10 words (and 10 objects, for the pairs task). From one mini-block to the next, the task alternated, and participants received a new set of words (and objects). In this way, the beginning of each mini-block should cause release from proactive interference in two ways: First, the change of task entailed a change of the kind of retrieval cue, from objects (in the pairs task) to ordinal positions (in the serial-recall task). Second, with the start of a new mini-block participants received a new set of retrieval candidates (i.e., the words), thereby eliminating the chance of confusing a word from the current memory set with words from preceding memory sets.

During the test phase, participants selected each to-be-retrieved word from the set of 10 candidates. We opted for this testing method because a first experiment of this series, in which we asked participants to type the words, revealed two drawbacks of that procedure. Besides recall being very poor, we observed opposite trends for item memory and binding memory over the trials within a mini-block: Item memory – the probability of recalling a word from the current memory set, regardless of whether it was the correct one – tended to increase across trials. By contrast, binding memory – the probability of recalling the correct word for the current cue, given that a list word was recalled at all – tended to decline over trials. Whereas the decline of binding memory could reflect the build-up of proactive interference, the increase of item memory could reflect participants’ learning of the candidate set of the mini-block. As the overall accuracy score – the probability of recalling the correct word in response to a cue – is the product of the item-memory and the binding-memory score, the two opposing tendencies canceled each other out. In this way, the effect of proactive interference on binding memory could be obscured by the growth of item memory from participants learning the candidate set. To avoid that, in the present experiments we presented participants with the candidate set, so that learning it over the course of a mini-block did not provide any additional knowledge that task performance could benefit from.

### Method

#### Participants

For each of the two experiments we recruited 50 native speakers of English (between 18 and 35 years old) on Prolific. We chose this sample size because we deemed it a good compromise between increasing the chance of obtaining unambiguous evidence and limiting the financial cost. As we planned to use Bayesian statistical inference, the choice of the initial sample size is not critical as we could decide to increase the sample size in light of ambiguous evidence to resolve the ambiguity (Rouder, 2014). It turned out that there was no need to do that in any of the experiments of the present series.

#### Materials

The pool of words consisted of 386 nouns referring to concrete objects taken from the data base of Devereux, Tyler, Geertzen, and Randall (2014). For each mini-block, 10 new nouns were sampled from that word pool as response candidates. For each serial-recall trial, seven words were sampled from the 10 candidates to form the memory set; for trials of the pairs task, six words were sampled in Experiment 1a, and five words in Experiment 1b. The objects were taken from the 2400 unique objects in Tim Brady’s collection of object pictures.^1^ For each mini-block using the pairs task, 10 new object pictures were sampled from that pool. For each trial six (Experiment 1a) or five (Experiment 1b) objects were sampled from the set of 10, and paired at random with the (six or five) words selected for that trial.

#### Procedure

**Figure 1:**
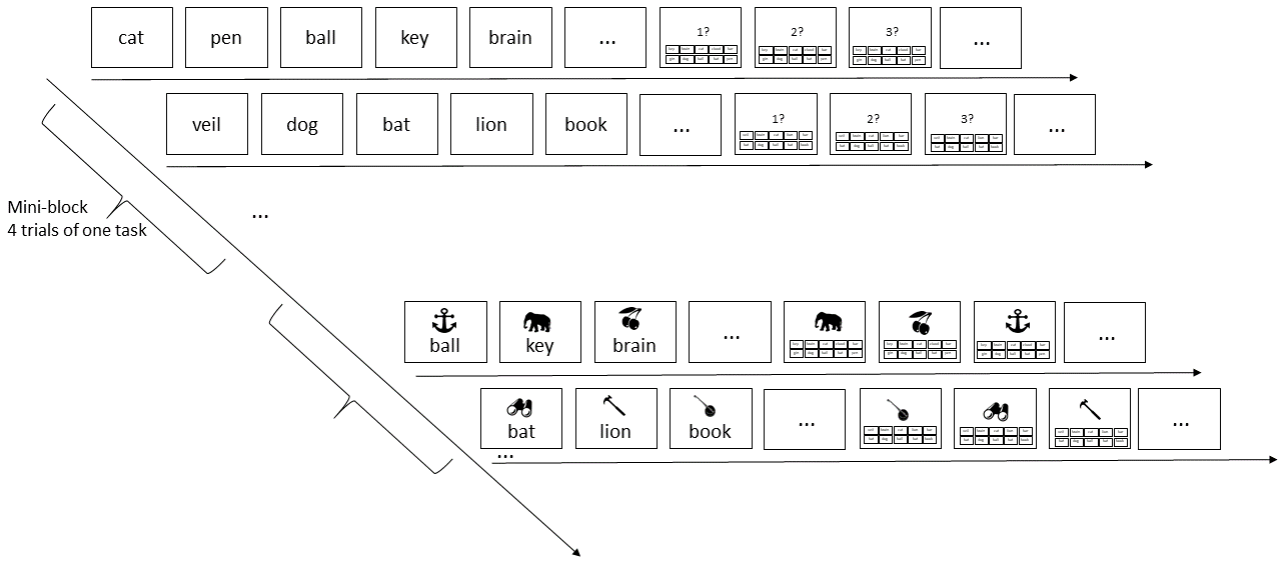
Illustration of the serial-recall task (top) and the pairs task (bottom). Each row shows the beginning of list presentation, and the beginning of the test phase, of one trial. Each mini-block consisted of four trials of the same task, followed by a mini-block with four trials of the other task.

The flow of events in a mini-block is illustrated in Figure 1. Each trial started with a fixation cross in the screen center for 1 s. In the pairs task, this was followed by the display of six (Experiment 1a) or five (Experiment 1b) object-word pairs in random order, each for 900 ms, followed by 100 ms blank screen. The object picture was presented horizontally centered in the top half of the screen, and the word was displayed below it in the bottom half of the screen, in black font on a gray background. Immediately after the offset of the last pair, the first object cue appeared in the top half of the screen, and the 10 words that served as response candidates were arranged in 2 rows of five in the bottom half. Participants selected the word they remember for the object cue with the mouse; upon selection the word briefly turned white. Then the next object cue was presented, and all candidate words turned black again, signaling that all 10 words could be chosen again for the next response. In this way, all six (or five) pairs were tested in a random order. In each trial, the candidate words were allocated to positions in the 2 × 5 array at random; their position remained constant for all tests of that trial. At the end of each trial, participants were asked to press the space bar to continue to the next trial.

In the serial-recall task, the seven words were presented in random order in the bottom half of the screen, in black font on a gray background, for 900 ms followed by 100 ms blank screen. Each word was accompanied by a digit representing its ordinal list position, displayed in red in the top half of the screen. After presentation of the last list word, the 10 response candidates were shown in the bottom half of the screen in the same way as for the pairs task, and the first retrieval cue – the digit 1 printed in red – was shown in the top half of the screen. Upon choosing a word from the candidate set, that word briefly turned white; then turned black again, and the next position cue (i.e., the digit 2) was shown. In this way, all list words were tested in forward order.

The experiment started with two mini-blocks for practice, followed by 12 mini-blocks of test trials. The task (pairs vs. serial recall) alternated regularly between the mini-blocks; the task of the first mini-block was counterbalanced across participants. The experiment took about 40 minutes to complete.

### Results

**Figure 2:**
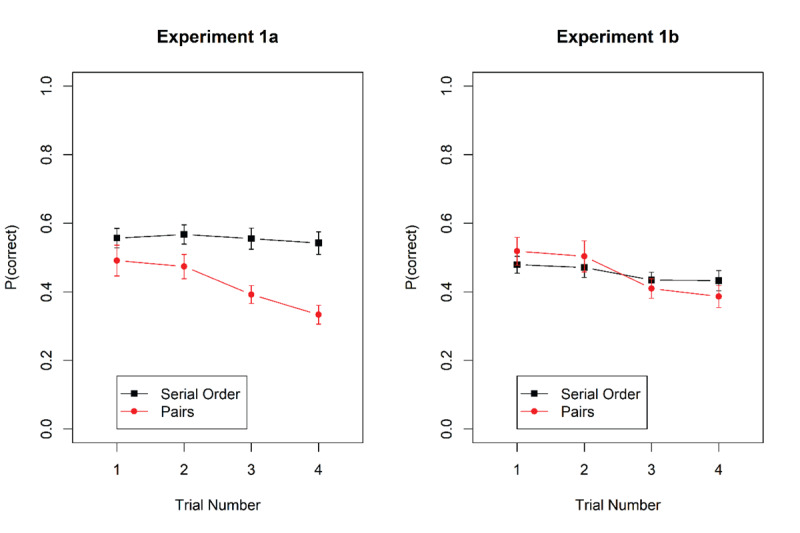
Proportion of correct responses in the serial-recall and the pairs task in Experiments 1a and 1b. Error bars are 95% confidence intervals for within-subjects’ comparisons (Bakeman & McArthur, 1996).

We scored performance as the proportion of correct words chosen on each trial. Figure 2 shows accuracy as a function of task and trial number within mini-blocks. Accuracy declined across trials for the pairs task but not for the serial-recall task.

We analyzed the number of correct responses in each trial through Bayesian logistic models run with the *brms* package (Bürkner, 2017) for R (R Core Team, 2020), with 8 chains of 20,000 iterations each. We assessed the strength of evidence for effects of interest through a series of model comparisons, using the bridge sampling algorithm (Gronau, Singmann, & Wagenmakers, 2018) for estimating the Bayes factor (BF), which reflects the relative strength of evidence in favor of one over another model in a pair-wise model comparison. We used z-standardized predictors, and Cauchy priors with scale 0.5 for fixed effects, which can be used as default priors for standardized effect sizes in logistic models (Oberauer, 2019).

We carried out a series of model comparisons, always comparing a model including the effect of interest (representing the alternative hypothesis) to an otherwise identical model excluding it (representing the null hypothesis). In these model comparisons, a BF_10_ > 1 reflects evidence in favor of the effect, whereas a BF_10_ < 1 reflects evidence against the effect (i.e., in favor of the null hypothesis). To express the strength of evidence in favor of the null hypothesis, we also report BF_01_ = 1/BF_10_. We started the model comparison with the full model including the fixed effects of task (pairs vs. serial recall), trial number in the mini-block (1 to 4), and serial position (1 to 6 or 5 for the pairs task, and 1 to 7 for the serial-recall task), and their interactions. The full model also included a random intercept, and random slopes for all main effects and two-way interactions. In a first step we tested whether all the random slopes were required to fit the data; as that was the case, we kept the full random-slope structure for all subsequent comparison steps (Oberauer, 2022a).

Table 2 summarizes the Bayes factors for all fixed effects. The result of primary interest is the unambiguous support for the interaction of task with trial number. We followed it up by separate tests for the main effect of trial number for each task. The effect of trial number received strong support for the pairs task, BF_10_ = 7.8 × 10^5^ and 4.0 × 10^5^ in Experiments 1a and 1b, respectively. By contrast, the null hypothesis was clearly supported for the serial-recall task in Experiment 1a, with BF_10_ = 0.07; BF_01_ = 14, and in Experiment 1b the support for a proactive-interference effect in serial recall was weak, with BF_10_ = 2.2.

**Table 2:** Bayes factors (BF_10_) in favor of fixed effects in Experiments 1a and 1b.


EFFECT	EXPERIMENT 1A	EXPERIMENT 1B

3-way interaction	0.05	0.15

Trial × Task	1.51 × 10^12^	1382

Trial × Serial Position	0.04	0.04

Task × Serial Position	1.68 × 10^95^	9.93 × 10^87^

Trial	3633	57907

Task	1706	0.10

Serial Position	1.33 × 10^10^	8.13 × 10^10^


### Discussion

For the pairs task, the gradual decline of performance over trials within a mini-block demonstrates proactive interference. This finding confirms our observation with a different experimental paradigm that has also shown that the pairs task suffers from proactive interference between trials at all but the smallest set sizes (Bartsch & Oberauer, 2023). By contrast, we obtained ambiguous evidence, or evidence against proactive interference, for the serial-recall task.

## Experiment 2

With Experiment 2 we investigated whether there is proactive interference in the probed-recall task. Like serial recall, probed recall is a test of memory for serial order, but it is more similar to the pairs task in that the list items are tested in a random order: Participants remember a list of words in their order of presentation, and are tested on each word by its serial position, given as a numerical cue, in random order. If the absence of proactive interference in serial recall has something to do with the fact that the list was reproduced in forward order, then we should see proactive interference between trials in the probed-recall task.

Experiment 2 was again run in two versions. Experiment 2a realized the same design as Experiment 1, with mini-blocks of four trials using the same task, and the same candidate set. In Experiment 2b we lengthened the mini-blocks to eight trials to investigate the possibility that proactive interference builds up over more than four trials. If that was the case, longer mini-blocks would provide stronger evidence for proactive interference, and we could perhaps detect it even in WM tasks that show no sign of proactive interference building up across four trials, such as the serial-recall task in Experiment 1.

### Method

#### Participants

Participants were 49 (Experiment 2a) and 60 (Experiment 2b) English native speakers between 18 and 35 years of age, recruited through Prolific.

#### Materials and Procedure

The words and objects were drawn from the same pools as for Experiment 1a, and the procedure for the pairs task was exactly the same, except that the set size was increased to seven pairs, because we anticipated that the probed-recall task – also with a set size of seven words – would be considerably harder than the serial-recall task, and its difficulty might be matched with that of the pairs task when run with the same set size. List presentation for the probed-recall task was identical to the serial-recall task in Experiment 1. At test, the digit cues indicating the tested position were presented in random order, and participants were instructed to select the word that they remember for the given ordinal position, rather than reproducing the list in forward order.

### Results

**Figure 3:**
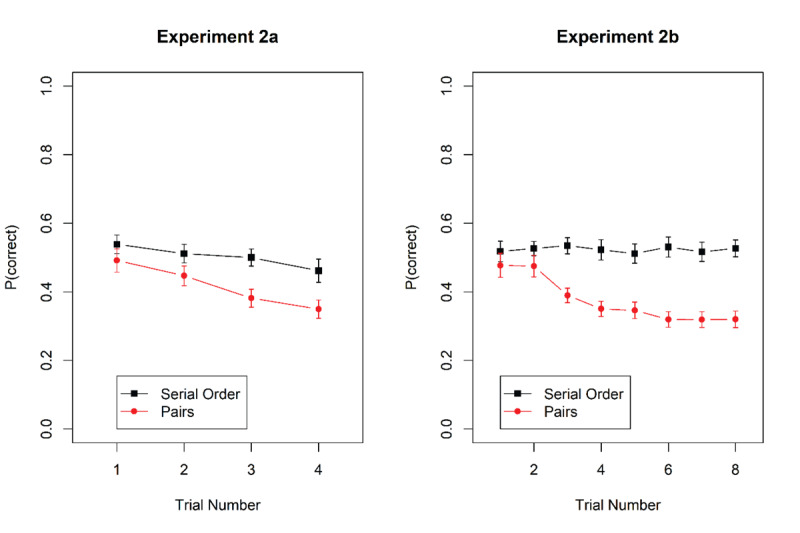
Accuracy in probed recall and pairs task as a function of trial within a mini-block in Experiments 2a and 2b. Error bars are 95% confidence intervals for within-subjects’ comparisons (Bakeman & McArthur, 1996).

Figure 3 shows accuracy as a function of trial number within mini-blocks for the two tasks. Table 3 summarizes the Bayes factors. In Experiment 2a, there was evidence for proactive interference in both tasks, and against an interaction of task with trial number. When analyzed separately, the main effect of trial number within a mini-block was supported for probed recall, BF_10_ = 28.7, and for the pairs task, BF_10_ = 3.59 × 10^5^. In Experiment 2b, however, there was a clear interaction between task and trial number. Testing the main effect of trial number separately for each task revealed evidence for proactive interference for the pairs task, BF_10_ = 1.66 × 10^11^, but evidence against proactive interference for the probed-recall task, BF_10_ = 0.041, BF_01_ = 24.5.

**Table 3:** Bayes factors (BF_10_) in favor of fixed effects in Experiments 2a and 2b.


EFFECT	EXPERIMENT 2A	EXPERIMENT 2B

3-way interaction	0.03	2.06

Trial × Task	0.19	7.94 × 10^8^

Trial × Serial Position	0.003	0.05

Task × Serial Position	0.2	391

Trial	5.00 × 10^6^	4.96 × 10^5^

Task	5064	2.92 × 10^11^

Serial Position	2154	60


### Discussion

Experiments 2a and 2b confirmed that the pairs task is vulnerable to proactive interference, and that effect was again larger than for a WM test of serial order, the probed-recall task. Whether probed recall is susceptible to proactive interference at all must remain open for now. Experiment 2b, which – by lengthening the mini-blocks and doubling the number of test trials – should be more sensitive to even a small effect of proactive interference, yielded evidence against it. Therefore, we find it more likely that the proactive-interference effect in Experiment 2a was a false positive, than that it is a real effect and Experiment 2b has missed it. We will come back to this question with Experiments 4 and 5. The data from the pairs task in Experiment 2b suggest that the build-up of proactive interference is largely complete after four trials, and therefore lengthening the mini-blocks beyond four appears unnecessary for detecting proactive interference when it is present. Therefore, we return to mini-blocks of four trials in the subsequent experiments.

## Experiment 3

With Experiment 3 we return to our search for proactive interference in the serial-recall task. We reasoned that perhaps our efforts to reduce cue-overload at the start of each mini-block by changing the retrieval cues between the serial-recall and the pairs task were not entirely successful. The pairs task still involved the sequential presentation of pairs, and therefore it is possible that the elements of each pair are not only associated to each other in eLTM, but also associated to their temporal or ordinal position within the trial. In that way, encoding of the pairs could have added to the cue overload of the temporal or positional cues that are used in the serial-recall task. To prevent that possibility, in Experiment 3 we replaced the pairs task by a task that avoided the sequential presentation of memory stimuli – a spatial reproduction task. It required participants to remember the locations of objects in a simultaneously presented array.

### Method

#### Participants

Participants were 50 native English speakers, between 18 and 35 years old, recruited through Prolific.

#### Materials and Procedure

**Figure 4:**
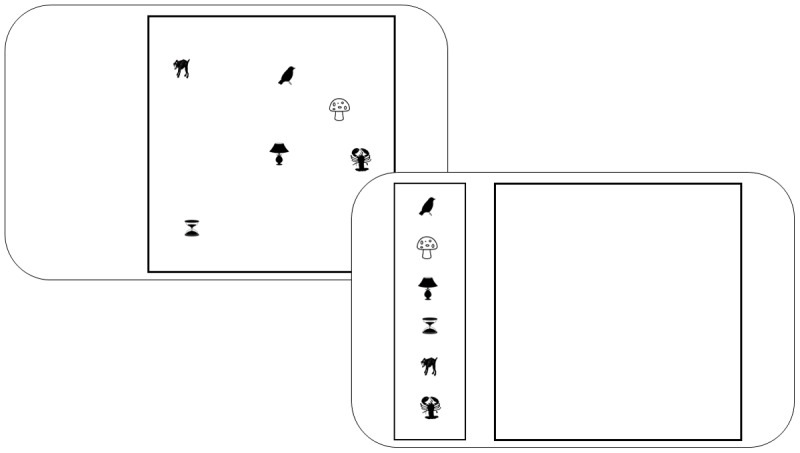
Illustration of the presentation screen (top left) and the test screen of the spatial reproduction task.

The serial-recall task was unchanged from Experiment 1a. The stimuli for the spatial reproduction task – illustrated in Figure 4 — were the objects from the pairs task in the preceding experiments. For each mini-block 10 new objects were sampled. In each trial, six of these objects were selected at random, and placed in random locations within a square frame centered horizontally on the screen, with a side length of approximately 90% of the screen height. The coordinates of each object were chosen at random with the constraint that the distance between any two objects was at least 1.5 times their diameter, which was set to about 8% of the screen height. The array of objects was presented for 3 s. After that, there was a 1 s retention interval during which only the empty frame was visible. For the test, the six objects were presented in a random order from top to bottom at the left of the square frame. Participants could drag each object with the mouse to its remembered location. They could correct each object’s location until they started moving the next object. Participants were instructed to move every object to its original location, but they did not have to move all objects. When satisfied with their reconstruction of the object locations, they ended the trial by clicking on a “confirm positions” button. Then they were invited to press the space bar to start the next trial.

### Results and Discussion

**Figure 5:**
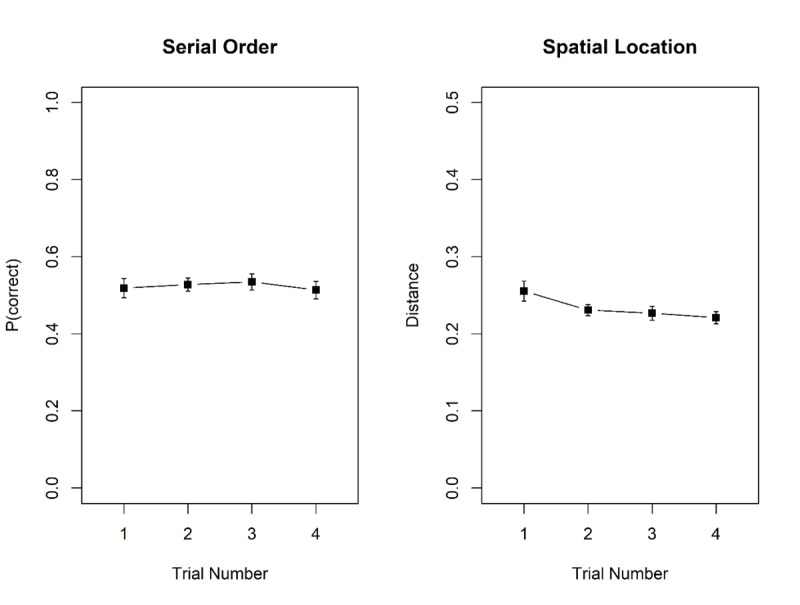
Proportion correct in the serial-recall task, and error of reproduction, measured as distance between the original and the reproduced location, in the spatial reproduction task, Experiment 3. Error bars are 95% confidence intervals for within-subjects comparisons (Bakeman & McArthur, 1996).

Performance in the spatial reproduction task was measured through the Euclidean distance between the original and the reproduced location of each dot, based on normalized coordinates (ranging from 0 to 1) in the square frame. Figure 5 shows memory performance in the two tasks as a function of trial number in the mini-block. There was no hint of proactive interference in either task. For serial recall, there was strong evidence against the effect of trial number, BF_10_ = 0.06; BF_01_ = 16.7. For the spatial reproduction task, the evidence supported an effect of trial number; BF_10_ = 19.1, but it signals an improvement of performance across trials, as reflected in a decrease of the average error of reconstruction.

To conclude, it is not the case that the sequential presentation of pairs in Experiment 1 prevented the release from proactive interference in the serial-recall task. Serial recall is not affected by proactive interference between trials.

## Experiment 4

With Experiment 4 we made another attempt at finding proactive interference in the probed-recall task, for which Experiments 2a and 2b left us with conflicting results. We followed the same rationale as with Experiment 3, replacing the pairs task – which potentially added to the cue overload of the temporal cues that are used in probed recall – by a color reproduction task. In color reproduction, participants remembered the colors in a simultaneously presented array, and tried to reproduce the color of all target items on a color circle. If probed recall of serially presented words is affected by proactive interference between trials, we should see that effect clearly, now that the interleaving task did not involve any temporal cues.

### Method

#### Participants

Participants were 33 native German speakers, between 18 and 35 years old, recruited from the student population of the University of Zurich for in-person participation in the lab.

#### Materials and Procedure

**Figure 6:**
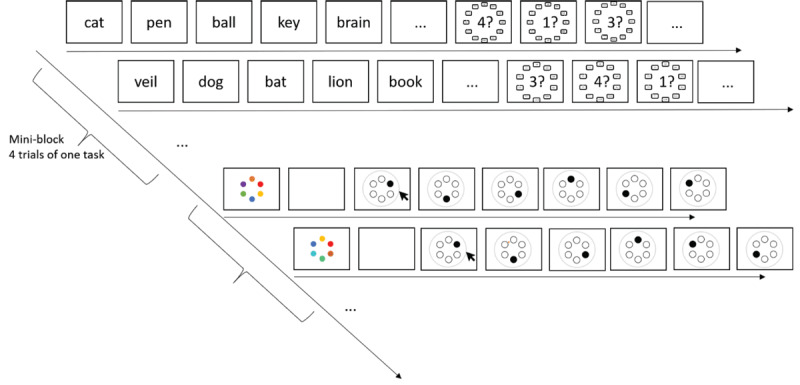
Task flow of the probed recall and color reproduction task of Experiment 4.

The tasks can be seen in Figure 6. The probed recall task entailed the sequential presentation of six words. After a 500 ms fixation cross that marked the beginning of a trial, words were presented centrally for 900 ms, followed by a blank 100 ms inter-stimulus-interval. Immediate memory was tested after a short retention interval of 500 ms. Memory for each item was cued by a digit denoting its serial position, in random order. The response options were 12 words, presented equidistantly on an invisible circle around the digit. The response options remained the same within a trial.

Different from the preceding experiments, here we sampled the words of each probed-recall trial without replacement from a large pool of 589 concrete German high frequency nouns (mean normalized lemma frequency = 24.23/million; drawn from the dlexdb.de lexical database). The response options included all words from the current list, one lure from each of the four immediately preceding trials of the probed-recall task^2^ (allowing for protrusion errors, i.e., erroneous choices of words from preceding trials), and two new words never seen before in the experiment. With this composition of memory lists and response options, we could test for a second indicator of proactive interference, in addition to the decline of performance over trials within a mini-block: Proactive interference from preceding trials should become manifest in a tendency to erroneously select words from recent preceding trials more often than new words.

In the color reproduction task, participants were presented with six discs colored with one of 360 colors sampled with replacement from a color wheel in the CIE L × a × b color space, centered on L = 70, a = 20, and b = 38, with a radius of 60. This is the color wheel most commonly used in continuous-reproduction tests of visual WM, because it consists of colors that are approximately equidistant in psychological similarity space, and approximately equally bright (for a critical discussion see Bae, Olkkonen, Allred, & Flombaum, 2015). The colored discs were presented uniformly in the chosen color against a white background. Immediate memory was tested using a continuous reproduction task.

We tested all six colors in random order. For each test the placeholder of the tested color was highlighted in black. The array of place holders was surrounded by a grey ring that covered the color circle from which participants were to select the color they remembered for the tested position. The colors behind the grey ring were rotated into a random orientation from trial to trial. A mouse arrow appeared in the center of the screen, and once participants moved the mouse away from the center in the direction of one of the (hidden) colors, the black circle assumed that color. Thus, by moving the mouse the participants continuously adapted the circle’s color. They were instructed to reproduce the color they remembered for the circle at that specific position as accurately as possible. They submitted their response by a mouse click. Performance in the color reproduction task was measured as the mean recall error, defined as the absolute angular deviation of the recalled color from the target.

The trials were organized into mini-blocks of four of the same task; tasks alternated between mini-blocks. There were 24 mini-blocks of test trials, preceded by 2 mini-blocks for practice.

### Results and Discussion

**Figure 7:**
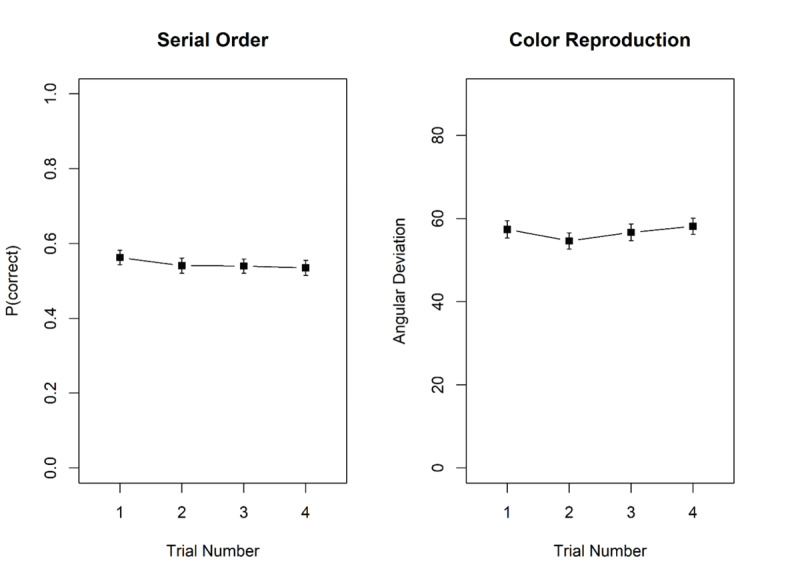
Proportion correct in the probed-recall task, and mean recall error, measured as angular distance between the original and the reproduced colour, in the colour reproduction task, Experiment 4. Error bars are 95% confidence intervals for within-subjects comparisons (Bakeman & McArthur, 1996).

Figure 7 shows memory performance in the two tasks as a function of trial number in the mini-block. There was no hint of proactive interference in either task. For probed recall, there was substantial evidence against the effect of trial number, BF_10_ = 0.17; BF_01_ = 5.84. Similarly, for the color reproduction task, the evidence against an effect of trial number was strong, BF_10_ = 0.03; BF_01_ = 38.5.

**Figure 8:**
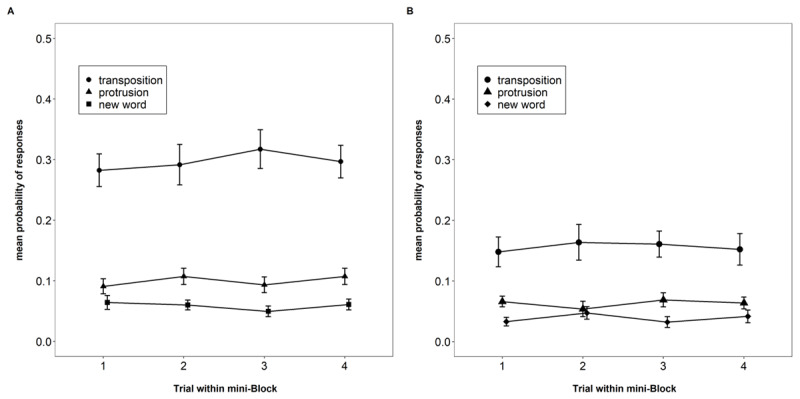
Proportion of each type of erroneous responses in the probed-recall task, Experiment 4 and 5 (Panel A and B, respectively). Error bars are 95% confidence intervals for within-subjects comparisons.

This experiment offers a second indicator of proactive interference for the probed recall task: We tested whether participants showed an increased tendency to erroneously select words from recent preceding trials over the course of a mini-block. As can be seen in Figure 8, this was not the case. Panel A shows that protrusions from previous trials occur barely twice as often as selections of new words, although the response set contained four protrusion lures and only two new lures. Their prevalence did not increase across trials of a mini-block.

To conclude, we found evidence against build-up and release from proactive interference in a test of serial order in WM – the probed recall task – as well as in a color reproduction task. This is in line with our findings of Experiment 2b and supports our interpretation that the small proactive-interference effect in Experiment 2a was a false positive. Taken together, probed recall is probably not susceptible to proactive interference, nor is the continuous reproduction of the colors of objects in a spatial array.

## Experiment 5

With Experiment 5 we tested the possibility that the formation of useful eLTM traces of memory lists takes longer than the common presentation time of one item per second. A hint that this might be the case comes from one previous experiment using a similar approach to test the effect of proactive interference on serial recall – presenting mini-blocks of five trials using the same material before switching to another – in which there was a small decline of performance across the trials of a mini-block, observed primarily with slow presentation rates (Oberauer, 2022b; Experiment 3). Therefore, with Experiment 5, we made a final attempt at finding proactive interference in the probed-recall task, by repeating Experiment 4 with a much slower presentation time for the word lists. If the formation of helpful eLTM traces takes longer than we allowed in the preceding experiments, we should now see the build-up of proactive interference across trials of a mini-block in the probed-recall task.

### Method

#### Participants

Participants were 29 native German speakers, between 18 and 35 years old, recruited from the student population of the University of Zurich for in-person participation in the lab.

#### Materials and Procedure

The task followed the procedure of Experiment 4 except for the change that the words in the probed recall task were presented for 900 ms, followed by a blank inter-stimulus interval of 4100 ms (vs. 100 ms in Experiment 4), yielding a presentation rate of one item every 5 seconds. The color reproduction task remained unchanged – giving us the opportunity to replicate the results of Experiment 4.

### Results and Discussion

**Figure 9:**
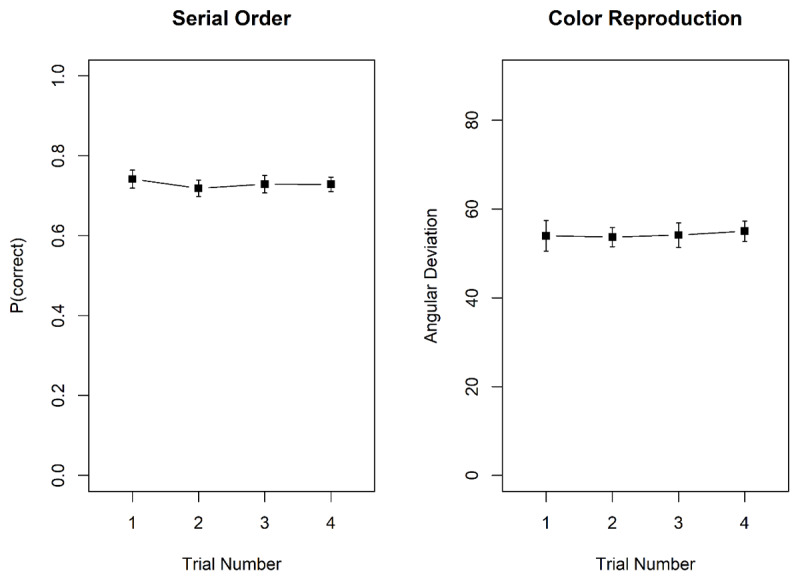
Proportion correct in the probed-recall task, and mean recall error, measured as angular distance between the original and the reproduced colour, in the colour reproduction task, Experiment 5. Error bars are 95% confidence intervals for within-subjects comparisons (Bakeman & McArthur, 1996).

Figure 9 shows memory performance in the two tasks as a function of trial number in the mini-block. As in Experiment 4, there was no hint of proactive interference in either task. For probed recall, there was strong evidence against the effect of trial number, BF_10_ = 0.06; BF_01_ = 15.51. Similarly, for the color reproduction task, the evidence against an effect of trial number was strong, BF_10_ = 0.03; BF_01_ = 33.92.

To conclude, we again found evidence against build-up and release from proactive interference in a test of serial order in WM – also with slower presentation rate – as well as in a color reproduction task. This is in line with our findings of Experiment 2b and 4.

## General Discussion

The distinction between working memory (or short-term, or primary memory) and long-term memory (or secondary memory) has a long history, and one full of debates. The distinction itself has been, and is still, contested (Nairne, 2002). Among those who accept it, there is no consensus about how and where to draw the line between these forms of memory, how strongly they are separated, and how they interact (Cowan, 2019; Foster, Vogel, & Awh, 2019; Norris, 2017). One implication of that uncertainty is the task-impurity problem. Whenever we try to probe a person’s WM, we face the possibility that their responses are – to an unknown part – determined by LTM, in particular episodic LTM. These contributions from eLTM could be responsible for any experimental effect that is interpreted as reflecting the mechanisms of WM, and it could be responsible for a large part of the variance of measured WM capacity in research on individual differences.

We leveraged proactive interference between trials to determine whether performance in several popular experimental paradigms for studying WM is in part reliant on eLTM. Fortunately for WM researchers, we found mostly evidence against proactive interference in tests of memory for serial order (i.e., serial and probed recall), and also in two tests of visual-spatial WM (i.e. reproduction of spatial locations and of color arrays). At the same time, Experiments 1 and 2 confirmed the observation of proactive interference in a WM test of memory for object-word pairs (Bartsch & Oberauer, 2023) using a different method for assessing proactive interference than the original work. We conclude that several work horses of WM research are not contaminated by substantial contributions of episodic memory, but some tasks used for investigating WM are.

### Heterogeneity of the Proactive Interference Effect in Serial-Order Memory

**Figure 10:**
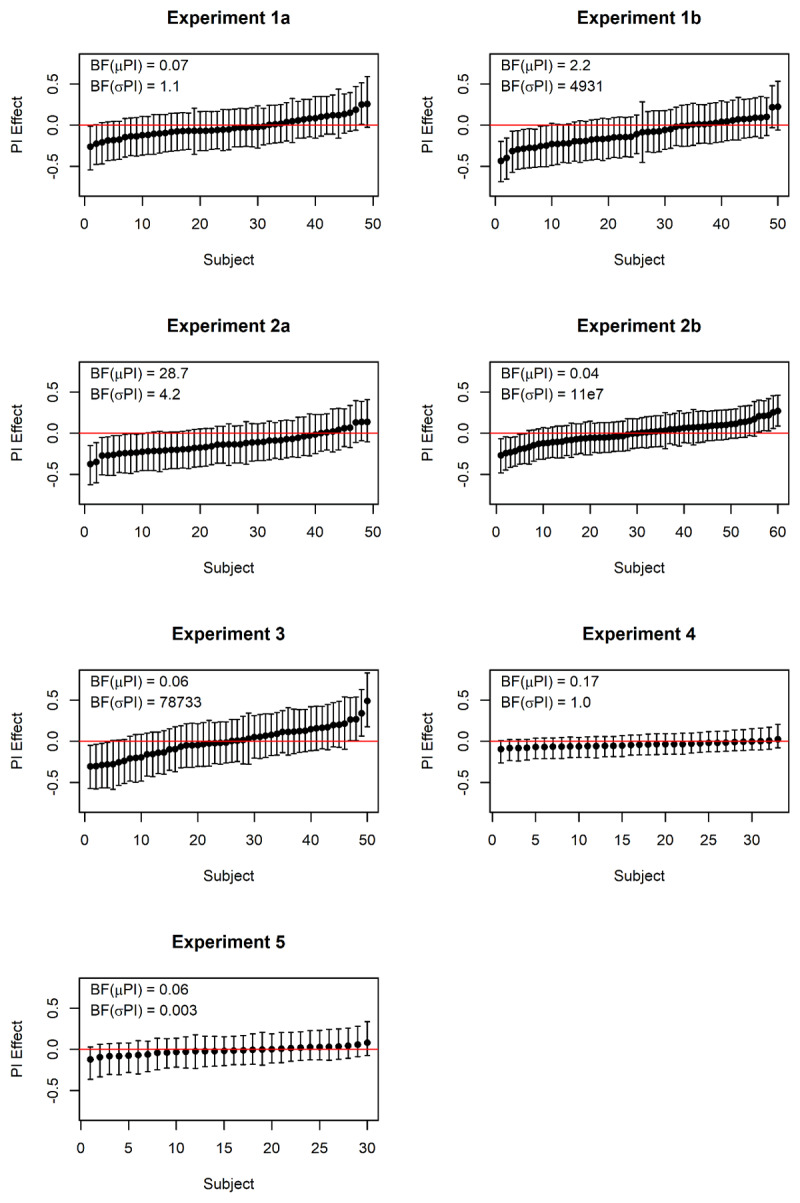
Posterior estimates of the effects of trial position within a mini-block for individual participants. Points represent the mean of the posterior, and error bars their 95% credible intervals. Negative effects reflect a decline of performance across trials in a mini-block, consistent with proactive interference. BF(μPI) is the Bayes factor in favor of including trial number as a fixed effect; BF(σPI) is the Bayes factor for including trial number as a random effect, that is, assuming individual differences in the effect.

Whereas the majority of our experiments yielded evidence against proactive interference in serial-order memory, a minority provided evidence in favor. Is that an inconsistency reflecting unidentified systematic differences between experiments? More likely, it reflects sampling error. Figure 10 presents the proactive-interference effects of individual subjects in the serial or probed recall condition of each experiment. These are estimates from the mixed-effects models, which separate individual differences from trial-to-trial noise, and therefore give a more accurate picture of individual differences than participants’ mean performance scores (Haaf & Rouder, 2019). The picture is essentially the same for all experiments: The large majority of subjects have credible intervals including zero. A few exceptions – in those experiments in which the BF supports the assumption of individual differences — are found at both the negative and the positive end of the scale. This probably means that trial number (i.e., proactive interference) does have an effect on performance for a minority of individuals, but that effect is positive for some, and negative for others. In some samples (such as Experiments 1b, 2a), the negative direction happens to predominate, resulting in evidence in favor of proactive interference.

### The Contents of Working Memory are Shielded Against Proactive Interference

Our conclusions about the involvement of eLTM in a task hinge on the assumption that the contents of WM are protected against proactive interference from earlier events that are no longer held in WM, but are still represented in episodic memory. This assumption goes back many decades (Wickens, Born, & Allen, 1963), and has been disputed by some researchers (Beaudry, Neath, Surprenant, & Tehan, 2014; Ralph et al., 2011). Recent work has corroborated the assumption that contents of WM are shielded against interference from eLTM by showing consistently that recall from WM is immune to proactive interference from previous trials as long as memory load does not exceed estimates of WM capacity (Bartsch & Oberauer, 2023; Oberauer & Awh, 2022; Oberauer et al., 2017; Oberauer & Greve, 2021). The present observation of several additional WM tasks that are immune to proactive interference even at set sizes severely challenging WM capacity provides further evidence for the conjecture that the contents of WM are not susceptible to interference from episodic-memory representations of preceding events.

The argument against WM being immune to proactive interference (Beaudry et al., 2014) mostly relies on the finding that in item-recognition tasks, lures that match a recent earlier trial are harder to reject than new lures (Atkinson, Herrmann, & Wescourt, 1974; Monsell, 1978). This effect reflects a familiarity signal, potentially from episodic memory, that lingers across trials and intrudes into the recognition decision. When WM is tested with a reconstruction method, as in the present experiments, this influence from eLTM plays no discernable role. There was no tendency in Experiments 4 and 5 to choose words from recent earlier trials with a higher probability than new words. This observation reinforces our conclusion that the contents of WM are protected against proactive interference. When there is an interfering effect of prior events, as in item recognition, it arises because the familiarity signal that the probe elicits in episodic memory intrudes in the decision process, and not because episodic-memory contents are admitted into WM and interfere with WM contents.

### When does episodic LTM contribute to tests of WM?

Our findings raise the question why tests of serial-order memory, as well as tests of spatial-location and color reproduction, are immune to proactive interference even when WM capacity is clearly stretched, whereas other tasks start showing proactive interference at a comparable level of memory load. One possibility is that with the former tasks, no episodic-memory trace is created for the to-be-remembered information. This possibility can be ruled out at least for serial recall because the Hebb repetition effect has been demonstrated with this task (Hebb, 1961; Page, Cumming, Norris, McNeil, & Hitch, 2013). In the Hebb repetition paradigm, participants work through a series of immediate serial-recall trials, and one list is repeated, typically in every third trial. Serial recall improves for the repeated list but not for the interspersed filler lists. The Hebb repetition effect demonstrates that a representation of the repeated list is formed in LTM. This is only possible if the first encounter of the repeated list already leaves a trace in LTM that is still available several trials later, because otherwise any subsequent repetition of that list would not be functionally different from a new list, and repetition could not lead to learning. The Hebb repetition effect has also been shown for a task testing memory for visual arrays, which differed from the color-reproduction task of our Experiments 4 and 5 only in that the colors were selected from a smaller set of distinct colors, rather than from the full color circle (Souza & Oberauer, 2022). Hence, it is very unlikely that no LTM trace is created for visual arrays like the ones used in Experiments 4 and 5.

If we assume that an episodic-memory trace is always created for a memory set in a WM test, there are two remaining explanations for why there is no proactive interference in some tasks even at high loads on WM capacity. One is that the traces in eLTM from a trial in these tasks are too weak, or too imprecise, to contribute substantially to performance in an immediate memory test. The other is that for some reason the WM system does not access these traces, even though they could contribute substantially to performance if accessed.

We have proposed elsewhere that the information flow between WM and LTM is controlled by a flexible gate (Bartsch & Shepherdson, 2022; Oberauer, 2009; Oberauer et al., 2017). The flexible-gate hypothesis emerged from considerations about the function of WM (Oberauer, 2009): One important function of WM is to provide a medium for building representations that can deviate from a person’s long-term knowledge (e.g., when constructing counterfactual scenarios) and habits (e.g., when programming a task set countermanding an action routine). As such, these representations need to be shielded against interference from LTM. To that end, the gate from LTM into WM is controlled such that, by default, the representations in WM guide thought and action. Yet, when a monitoring process comes to the result that relying on LTM representations instead is likely to achieve a better outcome, the gate is opened and the WM system retrieves available information from LTM to guide its computations.^3^

**Figure 11:**
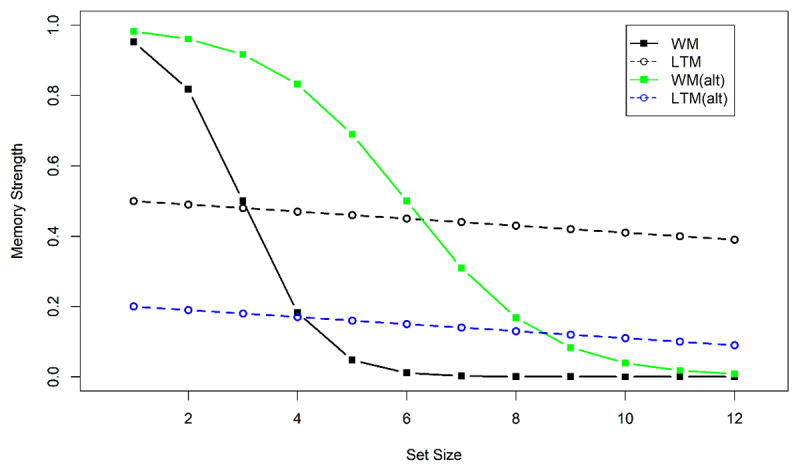
Schematic illustration of the flexible-gate hypothesis. Continuous lines show strength of WM representations, and broken lines the strength of eLTM representations. Black lines illustrate a task in which WM capacity imposes a severe constraint on memory strength, and episodic memory strength is high. An ideal gate-keeper would open the gate to eLTM when set size exceeds 3, because then relying on episodic memory leads to better performance than relying on WM. The green line shows an alternative scenario for a task where WM representations are less constrained by capacity. The blue line shows a scenario where episodic memory strength is poorer. Both alternative scenarios shift the point at which the gate should be opened to higher set sizes.

We can use the flexible-gate hypothesis as a conceptual frame for understanding the conditions under which eLTM contributes to performance in an immediate memory test, as illustrated in Figure 11. As set size increases, the memory strength^4^ of representations in WM degrades rapidly due to the capacity limit of WM. At small set sizes, episodic memory is weaker than WM for probably a multitude of reasons, proactive interference among them. However, eLTM has no capacity limit and therefore is more robust against increasing set size. Therefore, as set size is increased, there comes a point at which there is more information available in episodic memory than in WM. An optimal gate-keeping policy would be to open the gate when that point is surpassed. As a consequence, test responses are to a substantial degree based on episodic memory traces.

Figure 11 illustrates three ways in which WM tests can differ in the point on the set-size axis at which the WM system starts to recruit eLTM traces as the basis for responses, and hence, at which we should begin to observe proactive interference across trials. One is that set size has a less detrimental effect on information availability in WM in one task than another (green vs. black continuous line in Figure 11). Thereby the threshold for episodic traces being more available than WM traces is reached earlier for one task (black continuous crossing the dashed black line) than the other (green line crossing the dashed black line). For instance, Experiment 1a has shown that at the same set size, words are easier to retrieve when cued by spatial locations than when cued by objects, and that could explain why proactive interference was observed only when words were cued by objects. Experiment 2b, however, equated performance between the two tasks, and still only object-cued words suffered proactive interference. Therefore, this explanation alone cannot account for the results.

A second possibility is that for some tasks, episodic memory traces are more accessible or more precise than for others (black vs. blue broken lines). For instance, we could speculate that everyday objects are more powerful retrieval cues in episodic memory than ordinal list positions or spatial locations, because objects are more distinctive. Objects differ from each other on a multitude of feature dimensions, making them much easier to distinguish from one another than ordinal positions or locations, which differ on only one or two dimensions. This could explain why words cued by objects suffer proactive interference, whereas words cued by serial positions do not (Experiments 1 and 2), and why colors cued by objects suffer proactive interference (Oberauer & Awh, 2022), whereas colors cued by locations do not (Experiments 4 and 5). This idea, however, leaves unexplained why we see no proactive interference when objects serve as retrieval cues for spatial locations (Experiment 3). One way to explain that finding could be that the presentation time (500 ms. per object) was not long enough for useful eLTM representations of object-location relations to be formed. In Experiment 1 of Oberauer and Awh (2022) memory for the color of objects did not show any proactive interference when objects were presented at a rate of one every 200 ms, whereas there was proactive interference with a presentation rate of one object every 1000 ms. If the generation of eLTM traces is slower than that of WM traces, a faster presentation rate should substantially reduce the strength of eLTM, with a smaller impact on the strength of WM. As a consequence, the set size at which the gate should be opened for eLTM would shift to a higher value. If that is true, we should find substantial proactive interference for object-location memory when objects are presented one by one, for 1 s each, in their respective locations.

A third possibility to explain task differences in proactive interference with the flexible-gate hypothesis is that the WM system is biased to rely on episodic traces from LTM differently for different tasks. In the scenario with the two black lines in Figure 11, an optimal policy would be to start opening the gate and retrieving available eLTM traces for set sizes exceeding 3. If the gate control system were biased against eLTM for some tasks, it might start opening the gate at much higher set sizes (e.g., set size 7). That scenario leads to the prediction that memory performance declines with increasing set size up to the point where the gate is opened, and then increases again, because opening the gate now admits information from eLTM that enables much better performance than relying on WM alone. Such a non-monotonic set-size function has never been observed in any test of immediate memory. Therefore, the third explanation is extremely unlikely.

To conclude, we offer the following explanation of the present findings, based on the flexible-gate hypothesis. The flow of information between WM and eLTM is controlled by a gate. The decision whether to open the gate and admit episodic-memory information into WM is made on the basis of an assessment whether relying on eLTM is likely to result in a better response than relying on WM representations. That assessment is based on long-term experience with the quality and accessibility of relevant episodic-memory traces in the current situation, and a moment-to-moment metacognitive assessment of the quality of the current WM representations (Krasnoff & Oberauer, 2022; Rademaker et al., 2012). Proactive interference across trials is observed if two conditions are met. First, on a substantial proportion of trials the information that could be obtained from eLTM provides a better information basis for a response than the information in WM. High-dimensional retrieval cues such as everyday objects are more powerful cues in episodic memory than low-dimensional retrieval cues such as temporal context and spatial location, and therefore, that condition is already met at a much lower level of degradation of WM representations (i.e., earlier on the set-size scale in Figure 11). The second condition is that the mechanism controlling the gate represents the relative advantages of WM and eLTM representations accurately and with little bias.

This account could also help to explain an apparent paradox. Serial recall of verbal lists is not impaired by proactive interference. Yet, people occasionally recall an item from a list of an earlier trial, and when that happens, the item tends to be recalled close to its position in the original list (Fischer-Baum & McCloskey, 2015; Osth & Dennis, 2015). These so-called protrusions come from up to four trials back (Fischer-Baum & McCloskey, 2015), and therefore very likely come from eLTM. If protrusions from eLTM replaced correct responses that could have been given based on a representation of the current list in WM, then they should cause proactive interference in our experiments: They cannot occur in the first trial of a mini-block, but become increasingly likely towards the end of a mini-block. However, according to the flexible-gate hypothesis, episodic records of recent earlier trials don’t automatically intrude into WM. Rather, this information is admitted only when a meta-cognitive assessment process determines that the available representations in WM are insufficient for doing the task. This would occur on trials in which, for some reason (e.g., a lapse of attention, or a particularly difficult list composition) the list representation in WM is particularly poor. In these cases, drawing on episodic memory could hardly make performance worse – it could make it better (if the episodic information matched the current list) or replace an error based on poor WM representations by an error relying on episodic memory of an earlier list – that is, a protrusion (see Oberauer et al., 2017; for an analogous explanation of an analogous paradoxical constellation of findings).

## Conclusions

There is good news and bad news. The bad news is that some tasks that the WM research community uses to investigate WM are severely contaminated by eLTM – among them, cued recall of conjunctions of everyday objects with words, or with colors. The good news is that many of the most popular paradigms, including serial and probed recall, and the standard version of the continuous color-reproduction task, are not. Part of the bad news is that we have not yet identified general rules for determining under which conditions a test of immediate memory does or does not reflect a substantial contribution of eLTM. On the side of the good news, there is a method for determining the degree to which that is the case, and the present experiments contribute to demonstrating its usefulness: If a test of immediate recall is immune to proactive interference across trials, then its contamination by episodic memory is probably negligible.

## Data Accessibility Statements

All data reported in this article are available on the OSF: osf.io/yrpf6.

## Footnotes

https://bradylab.ucsd.edu/stimuli.html.

For trials that did not have 4 preceding trials in the same mini-block, the lures were drawn from the trials of the preceding mini-block of the probed-recall task (e.g. for trial 2, the –3 and –4 lures were from the last two trials of the previous probed-recall mini-block. For the very first mini-block of the experiment, new words were given as response options.

This monitoring process must rely on meta-cognitive knowledge about the quality of WM representations. There is evidence that people have such knowledge on a trial-by-trial basis (Arnicane, Oberauer, & Souza, 2021; Rademaker, Tredway, & Tong, 2012), and they can even order the report of items within a trial by their quality (Adam, Vogel, & Awh, 2017).

We understand *memory strength* as a short-hand for some combination of quality and availability of a memory representation that determines performance in a given test situation.
